# Functional Heterogeneity within the Primate Ventral Striatum for Motivational Regulation

**DOI:** 10.1523/JNEUROSCI.2430-24.2025

**Published:** 2025-04-14

**Authors:** Haruhiko Iwaoki, Yukiko Hori, Yuki Hori, Koki Mimura, Kei Oyama, Yuji Nagai, Toshiyuki Hirabayashi, Ken-ichi Inoue, Masahiko Takada, Makoto Higuchi, Takafumi Minamimoto

**Affiliations:** ^1^Advanced Neuroimaging Center, National Institutes for Quantum Science and Technology, Chiba 263-8555, Japan; ^2^National Institute of Neuroscience, National Center of Neurology and Psychiatry, Tokyo 187-8551, Japan; ^3^Systems Neuroscience Section, Center for the Evolutionary Origins of Human Behavior, Kyoto University, Aichi 484-8506, Japan

**Keywords:** checking, monkey, motivation, muscimol, resting, ventral striatum

## Abstract

The ventral striatum (VS) is a key brain region for reward processing and motivation, and its dysfunctions have been implicated in psychiatric disorders such as apathy and obsessive–compulsive disorder. Although functional heterogeneity within the VS has been well established in rodents, its relevance and mechanisms in primates remain unclear. To address this issue, we performed bilateral pharmacological inactivation of the VS in two male macaque monkeys using muscimol, a GABA_A_ receptor agonist. Precise targeting was achieved through computed tomography and magnetic resonance imaging. Behavioral effects were evaluated using two methods: a goal-directed task with variable rewards and analysis of spontaneous behavior. Our results demonstrated that anterior (a)VS inactivation induced a hypoactivity state that we termed “resting,” whereas posterior (p)VS inactivation elicited compulsive-like “checking” behaviors. Notably, neither the aVS nor the pVS inactivation affected reward value or drive processing, thus differentiating aVS and pVS from those involved in incentive motivation, such as the rostromedial caudate and ventral pallidum. Retrograde tracing demonstrated distinct anatomical projection patterns for the aVS and pVS, supporting their functional segregation. Together, the present results suggest the functional heterogeneity of the primate VS along its anterior–posterior axis, with the aVS and pVS participating in distinct motivational control circuits. Our findings may have important implications for understanding the neural mechanisms of psychiatric disorders and for the development of new therapeutic approaches.

## Significance Statement

The ventral striatum (VS) is a core brain region that is involved in motivation and reward-based behaviors. Its dysfunction is implicated in psychiatric disorders such as apathy and obsessive–compulsive disorder. In macaque monkeys, we used imaging-guided pharmacological manipulations to reveal that the anterior (aVS) and posterior VS (pVS) subregions have distinct roles in motivation, independent of the incentive or reward drive. Specifically, aVS inactivation induced a hypoactive state, whereas pVS inactivation elicited compulsive-like behaviors. These findings reveal distinct motivational mechanisms within the primate VS, thus offering valuable insights into the neural basis of psychiatric disorders and identifying promising therapeutic targets.

## Introduction

Motivation is a psychological process that directs, initiates, and sustains behavior toward a goal (Atkinson, 1964; Dickinson and Balleine, 1994). Disruptions in motivational control are associated with various mental health disorders such as apathy where the initiation and persistence of behaviors are impaired; obsessive–compulsive disorder (OCD) where individuals become excessively motivated toward maladaptive behaviors (Levy and Dubois, 2006; Figee et al., 2011; Gillan and Robbins, 2014). Accordingly, an understanding of the neural mechanisms of motivational control is critical from both biological and clinical perspectives. This is especially important in nonhuman primates, whose brain anatomy, function, and behavioral repertoire share significant similarities with those of humans.

The ventral striatum (VS) is a core part of the “reward circuit” and plays an important role in motivational control because of its extensive anatomical connections with limbic cortical and subcortical areas (Haber and McFarland, 1999; Haber and Knutson, 2010). In monkeys, neuronal activity within the VS has been shown to signal various aspects of reward processing, including magnitude, prediction, omission, timing of acquisition, and reward-driven motivation (Schultz et al., 1992; Bowman et al., 1996; Hollerman et al., 1998; Shidara et al., 1998; Tremblay et al., 1998; Cromwell and Schultz, 2003; Nakamura et al., 2012). Complementary human neuroimaging studies have revealed that activity in the VS correlates with both the amount of reward offered and the effort required to obtain a reward (Knutson et al., 2001; Pessiglione et al., 2007). These findings suggest that the VS in primates is crucial for both reward-related information processing and motivational control.

However, motivational control extends beyond reward-seeking behaviors, and growing evidence suggests that the VS also regulates nonreward behaviors. In rodents, for example, the optogenetic activation of neurons in the nucleus accumbens (a structure within the VS) increases self-grooming behaviors (Zhang et al., 2021). In monkeys, hypoactivity has been observed following the local activation of the VS (Worbe et al., 2009), further suggesting that this region contributes to a broad range of motivational processes. Moreover, experimental lesions of the primate VS do not directly impair reward behavior (Stern and Passingham, 1996), and lesion-induced effects are limited compared with other regions, such as the amygdala (Costa et al., 2016). These findings highlight serious limitations to our current understanding of the specific roles of the primate VS in motivational control.

Given the involvement of the VS in both spontaneous and reward-driven behaviors, the behavioral consequences of VS manipulation need to be explored from multiple perspectives. Specifically, regarding goal-directed behavior, classic psychological models suggest that motivation is influenced by two factors: the incentive value of rewards and drive (Hull, 1943; Spence, 1956; Toates, 1986). These factors should be evaluated separately when studying how the VS governs goal-directed behaviors. In addition, research in rodents has revealed that focal inactivation of the nucleus accumbens along its anterior–posterior axis elicits opposing reactions, appetitive eating, and defensive treading (Reynolds and Berridge, 2001). However, the relatively deep location of the VS in the primate brain poses challenges for identifying region-specific functions, which has resulted in a substantial gap in our understanding of the VS functions between primates and rodents.

Here, we investigated the behavioral effects of VS inactivation in macaque monkeys through the local injection of muscimol (a GABA_A_ receptor agonist) in both goal-directed and free-moving behavioral contexts. For the goal-directed task, we used a motivational paradigm that allowed us to distinguish the effects of incentive and drive on motivation for action. Given that the limbic system generally exerts similar functions across both hemispheres and lacks clear lateralization, unilateral manipulation may produce compensatory effects. We therefore targeted mirror-symmetric regions of the VS precisely under the guidance of computed tomography (CT) and magnetic resonance (MR) imaging. Together with complementary anatomical tracing data, our findings revealed functional differences within the primate VS in motivational control. These results suggest potential implications for the underlying mechanisms of psychiatric conditions associated with motivational dysregulation and offer novel approaches to their treatment.

## Materials and Methods

### Subjects

Three male rhesus monkeys (*Macaca mulatta*; Monkey RI, 6.3 kg; Monkey BI, 8.0 kg; Monkey #250, 6.1 kg) were used. Monkeys RI and BI were previously used in an inactivation study targeting the rostromedial caudate nucleus and ventral pallidum (Nagai et al., 2016; Fujimoto et al., 2019). All experimental procedures followed the Guide for the Care and Use of Nonhuman Primates in Neuroscience Research (The Japan Neuroscience Society; https://www.jnss.org/en/animal_primates) and were approved by the Animal Ethics Committee of the National Institutes for Quantum Science and Technology (#11-1038). Food was available *ad libitum*, and motivation was controlled by restricting access to fluid before experimental sessions in which water was provided as a reward for task performance. Animals received water supplementation whenever necessary (e.g., when they were unable to obtain sufficient water through experimentation) and had free access to water whenever testing was interrupted for >1 week. For environmental enrichment, play objects and/or small food items (fruit, nuts, and vegetables) were provided daily in the home cages.

### Surgery

Three monkeys underwent surgery under general isoflurane anesthesia (1–2%) for the implantation of either one or two chambers and a head fixation device (for Monkeys BI and RI) or before receiving a viral vector injection (for Monkey #250) for the retrograde tracing study (detailed below). For Monkey BI, a single chamber (22 × 22 mm ID; K.D.S.) was placed vertically, whereas for Monkey RI, two chambers (19 mm ID; Crist Instrument) were placed at a 20° angle from the coronal plane. Prophylactic antibiotics and analgesics were administered after surgery.

Prior to surgery, the stereotaxic coordinates of target brain structures were estimated using overlaid MR and CT images created using the PMOD image analysis software (PMOD Technologies). The CT scans (Accuitomo170, J. Morita) and MR imaging (7 T, NIRS/KOBELCO/Bruker or BioSpec 70/40, Bruker) were performed under anesthesia (continuous intravenous infusion of propofol, 0.2–0.6 mg/kg/min, i.v.).

### Muscimol microinjection

**Figure 1.**
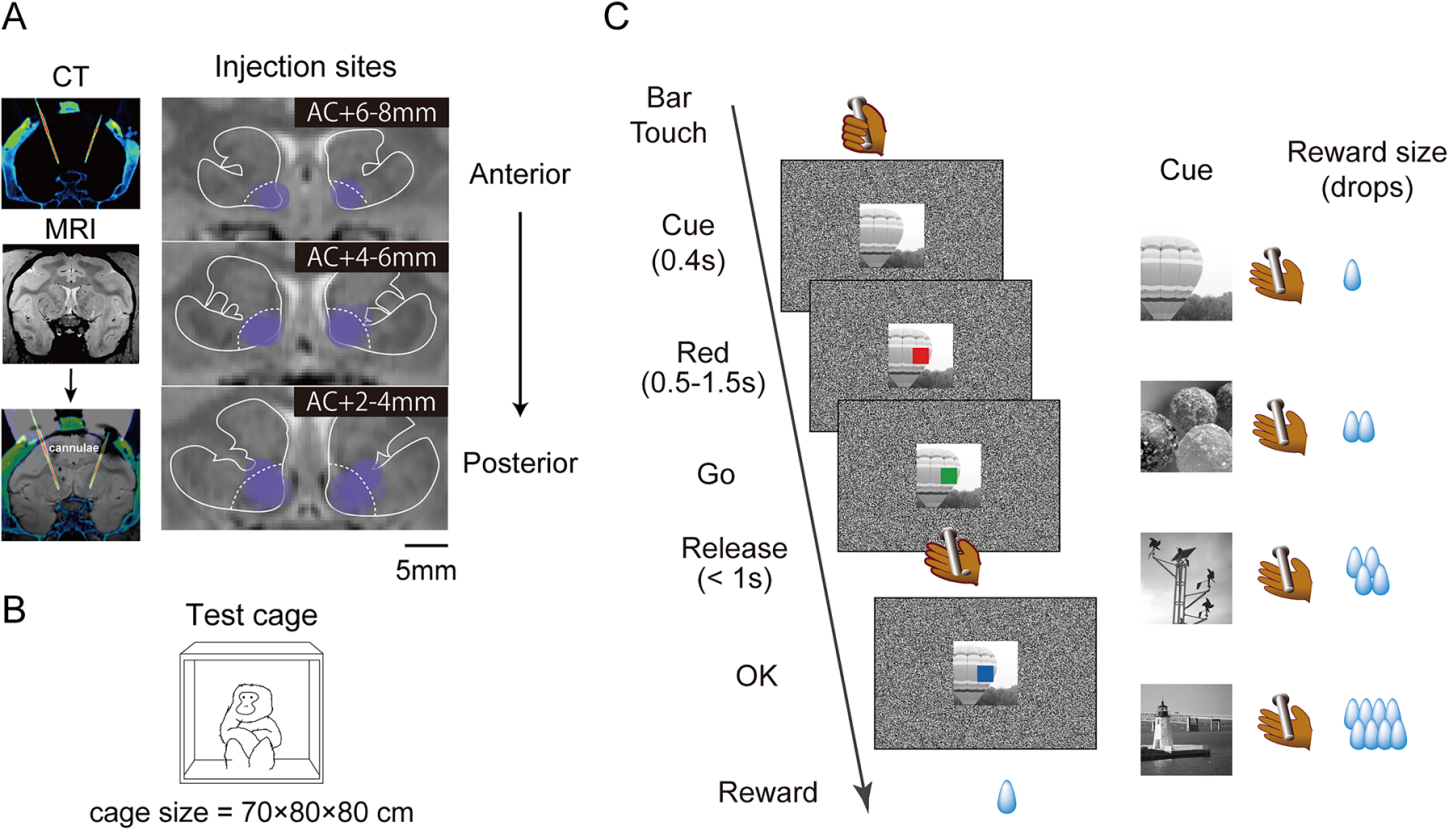
Experimental procedures. ***A***, Localization of injection sites using CT and MR imaging. Left, CT image visualizing the injection cannulae targeting the bilateral VS (hot color) overlaid on the MR image (grayscale) from Monkey RI. Right, Muscimol injection sites, with each purple circle indicating an estimated muscimol diffusion area (∼3 mm) from the tip of cannula marked on an MR image from Monkey BI. Dotted lines indicate VS boundaries within the striatum. “AC+” indicates the anterior distance from the center of the anterior commissure. ***B***, Illustration of the test cage environment for free-moving behavior. During the session, each monkey was isolated within the cage for observation and recording. ***C***, Reward-size task sequence. Left, Trial sequence. Each trial began when the monkey gripped a bar mounted at the front of the chair. If the monkey continued to grip, a black-and-white image (“cue”) and a colored square appeared on the screen. Upon the appearance of a green square (“go” signal), the monkey was required to release the bar within 200–1,000 ms to receive a liquid reward. If the monkey released the bar before the “go” signal or held the bar for longer than 1 s, the trial was marked as an error, and no water reward was provided. A correct release turned the screen spot blue (“correct” signal). Right, Reward contingency. A reward of 1, 2, 4, or 8 drops of water (1 drop, ∼0.12 ml) was delivered immediately after the correct signal. Each reward size was selected randomly with equal probability, and the cue presented at the beginning of the trial indicated the reward amount for that trial.

The GABA_A_ agonist muscimol (M1523, Sigma-Aldrich) was injected bilaterally and mirror-symmetrically into the VS to inactivate neuronal activity, following previously reported procedures (Nagai et al., 2016). Guide tubes were inserted through a grid hole in the implanted injection chamber, and stainless steel cannulae (outer diameter 300 µm; Muromachi) were advanced using a microdrive (MO-97A, Narishige). Muscimol (3 µg/1 µl saline) was injected at a rate of 0.2 µl/min using an auto-injector (Legato210, KD Scientific) to simultaneously deliver a total volume of 2 µl per side. In the saline control sessions, the same amount of saline was injected. The locations of injection cannulae were visualized using CT scans before or after the behavioral tests, and tip locations were mapped onto MR images using the PMOD image analysis software (Fig. 1*A*). Saline control injections and sham injections (guide tubes inserted without any injection) were used as controls. The muscimol or control injections were performed once per week.

### Experimental design

The behavioral effects of local VS inactivation were assessed in two contexts: spontaneous behaviors in a test cage and goal-directed behaviors in a motivational task. Monkey RI underwent either the free-moving test or the motivational task in a single experimental session, with four exceptions (Extended Data Table 1-1). On the day of the free-moving test, a CT scan was conducted to visualize the location of the injection cannulae (∼10 min), followed by muscimol or saline injection (∼10 min). After a 30 min waiting period, the monkey was placed in the test cage for 60 min of behavioral observations. On the day of the motivational task, the task (∼100 min) was conducted after the injection, followed by a CT scan. Monkey BI underwent both tests after all muscimol injection sessions and in three control sessions in the following order: injection (10 min), motivational task (100 min), CT scan (10 min), and free-moving test (60 min). The other three control sessions were conducted similarly to those of Monkey RI. The detailed injection sites and experimental conditions are listed in Extended Data Table 1-1.

### Spontaneous behaviors

Spontaneous behaviors were assessed in an isolated test cage for 1 h (Fig. 1*B*). For Monkey RI, the test cage was its home cage, which was located out of sight of other monkeys. Monkey BI was tested in a room with no other monkeys present. Monkey behavior was recorded at 30 fps using a video camera (RealSense D435, Intel) positioned in front of the cage. Monkeys were habituated to the recording environment for 2–3 weeks prior to testing. During recording, the water bottle and feeding box were removed, and the cage was illuminated using light-emitting diode lights.

### Goal-directed behavior

Each monkey was seated in a primate chair in a sound-attenuated dark room for the behavioral training and testing. Visual stimuli were presented on a computer video monitor placed in front of the monkey. Behavioral control data and data acquisition were performed using a real-time experimentation system (Hays et al., 1982), and visual stimuli were displayed using the Presentation software (Neurobehavioral Systems). In the reward-size task (Fig. 1*C*), the monkey had to release a bar to obtain liquid rewards. Trials began when the monkey touched the bar at the front of the chair. After a visual cue and a red target (the wait signal) appeared on the monitor, the target turned green following a variable interval (0.5–1.5 s). The monkey then had to release the bar between 0.2 and 1 s to receive a liquid reward (1–8 drops of water; 1 drop, ∼0.12 ml). In each trial, the visual cues were randomly changed. An intertrial interval of 1 s was enforced before the next trial began. If the monkey released the bar before the green target appeared, released the bar within 0.2 s after it appeared (early release), or failed to release the bar within 1 s (late release), the trial was terminated immediately and was repeated after the 1 s intertrial interval. Before each testing session, monkeys were subject to ∼22 h of water restriction without any behavioral testing. Each testing session continued for 100 min.

### Statistical analysis

In the free-moving context, spontaneous behaviors were categorized into the five most observed behaviors: “standing” (standing up on two legs), “resting” (sitting with head down and motionless), “grooming” (self-grooming), “checking” (manipulating the corners of the cage with fingertips), and “biting” (biting the chain of the collar). To quantify these behaviors, we implemented an object detection deep learning algorithm [You Only Look Once (YOLO) v5; https://github.com/ultralytics/yolov5] to analyze postural patterns in the video recordings on a frame-by-frame basis. To minimize redundancy and enhance generalizability, 100 representative frames were extracted from each session using a *k*-means frame selection method implemented in DeepLabCut 2.1 (Mathis et al., 2018). An expert experimenter familiar with monkey behaviors manually annotated each frame by drawing a bounding box around the monkey and labeling it with one of the five behavioral categories when applicable. Separate YOLO models were trained for each monkey using 80% of the annotated frames for training and 20% for testing. The models achieved mean average precision scores of 0.87 for Monkey RI and 0.84 for Monkey BI. These trained models were then applied to automatically classify behaviors across all video frames in each session. For each session, the number of frames classified into each behavioral category was counted, and the relative proportion of each behavior was calculated. For data-driven clustering based on the five characteristic behaviors, Ward's hierarchical clustering method (Ward, 1963) with Euclidean distance was applied to the behavioral data (maximum number of clusters, 10). To statistically assess the regional differences in the expression of “checking” and “resting” behaviors, we conducted *χ*^2^ tests separately for each monkey.

For the reward-size task, error rates in task performance were calculated by dividing the total number of errors by the total number of trials for each reward size and were then averaged across all sessions. Error rates were fitted to an inverse function of reward size, *E* *=* 1/aR, where R is the reward size, a is a constant parameter for all individual subjects, and *E* is the error rate (%) of the monkeys in trials with reward size R. The details were as follows (as reported in Minamimoto et al., 2009). We performed repeated-measure analysis of variance (ANOVA) with subjects as a random effect to examine the effect of treatment × reward size on error rate as well as the effect of treatment on the total number of errors and trials, rewards earned, and average reaction times in each session. Post hoc comparisons were made using Tukey's honestly significant difference test, with statistical significance of 0.05. The data of rostromedial caudate and ventral pallidum inactivation were reanalyzed from data originally obtained by Nagai et al. (2016) and Fujimoto et al. (2019), respectively.

### Retrograde tracing study

In Monkey #250, retrograde tracing was performed using adeno-associated virus 2-retro (AAV2retro) vectors expressing fluorescent proteins. Injections targeted the anterior (a)VS (AAV2retro-hSyn-mScarlet) and posterior (p)VS (AAV2retro-hSyn-AcGFP). The AAV titer was 2.0 × 10^13^ particles/ml, and the volume was 1 µl. The injections were performed with the assistance of the Brainsight Vet Robot System (VRCT002, Rogue Research). The intraoperative localization of injection cannulae was navigated using Brainsight (Rogue Research) based on overlaid images of preoperative MRI and CT data. The vectors were pressure-injected using a 10 µl syringe (Model 1701RN, Hamilton) with a 30 gauge injection needle placed in a fused silica capillary (outer diameter, 450 µm), which minimized backflow by creating a 500 nm space surrounding the needle tip. The microsyringe was mounted into a motorized microinjector (UMP3T-2, WPI) that was held by the robot arm. After a burr hole (8 mm in diameter) and a hole in the dura mater (∼5 mm in diameter) were made, the injection needle was inserted into the brain and slowly moved down to 2 mm beyond the target. It was maintained stationary for 5 min before being pulled up to the target location. The injection speed was set at 0.25 µl/min. After the injection, the needle remained in situ for 15 min to minimize backflow along the needle. Additional surgical procedures are outlined above, Surgery.

### Histology and image acquisition

Following a survival period of 34 d, Monkey #250 was immobilized using ketamine (10 mg/kg, i.m.) and xylazine (0.5 mg/kg, i.m.), deeply anesthetized with an overdose of sodium thiopental (50 mg/kg, i.v.), and then transcardially perfused with saline at 4°C followed by 4% paraformaldehyde in 0.1 M phosphate-buffered saline, pH 7.4. The brain was removed from the skull, postfixed in the same fresh fixative overnight, and saturated with 30% sucrose in a phosphate buffer at 4°C. Coronal sections (50 µm) were then cut serially using a freezing microtome. For double immunofluorescence to detect green fluorescent protein (GFP) and red fluorescent protein (RFP), the sections were blocked in 1% skim milk at room temperature for 1 h. They were then incubated for 2 d at 4°C in a mixture of rabbit anti-GFP monoclonal antibody (1:1,000 dilution; Invitrogen) and rat anti-RFP monoclonal antibody (1:1,000 dilution; Proteintech) in 0.1 M phosphate-buffered saline containing 2% normal donkey serum and 0.1% Triton X-100. The sections were subsequently incubated for 2 h at room temperature with a cocktail of Alexa Fluor 488-conjugated donkey anti-rabbit IgG antibody (1:400 dilution; Thermo Fisher Scientific) and Alexa Fluor 555-conjugated donkey anti-rat IgG antibody (1:400 dilution; Thermo Fisher Scientific). Images of the stained sections were then captured using a digital slide scanner (Nano-Zoomer S60, Hamamatsu Photonics K.K.; 20× objective, 0.46 µm per pixel) or a microscope equipped with a high-grade charge–coupled device camera (Biorevo, Keyence). The images were imported into a personal computer as digital data, and fluorescent protein expression was confirmed by enlarging the images. The locations of labeled neurons were plotted onto a macaque atlas (Dubach and Bowden, 2009; Rohlfing et al., 2012, https://scalablebrainatlas.incf.org/macaque/DB09) for the anatomical analysis.

## Results

We locally inactivated the bilateral VS by injecting muscimol, a GABA_A_ receptor agonist, across 29 sessions in two monkeys (6 and 23 sessions for Monkeys BI and RI, respectively). We examined changes in spontaneous behavior in 13 muscimol sessions (six and seven sessions for Monkeys BI and RI, respectively) and compared them with those in 10 control sessions (three and seven sessions, respectively). We also examined behavioral changes in a goal-directed task during 26 muscimol sessions (6 and 20 sessions for Monkeys BI and RI, respectively) and compared them with those in 16 control sessions (6 and 10 sessions, respectively). The details of the injection conditions and locations are summarized in Extended Data Table 1-1 and illustrated in Figure 1*A*.

### Effects of VS inactivation on spontaneous behaviors

To examine the effects of VS inactivation on spontaneous behaviors, we isolated each monkey in its cage for 1 h to minimize social interactions (Fig. 1*B*). The monkeys showed five characteristic behaviors during the experiment. In control sessions, the predominant behaviors were “standing” and “grooming” for both monkeys. However, VS inactivation induced two atypical behaviors in both monkeys: “resting” and “checking.”

**Figure 2.**
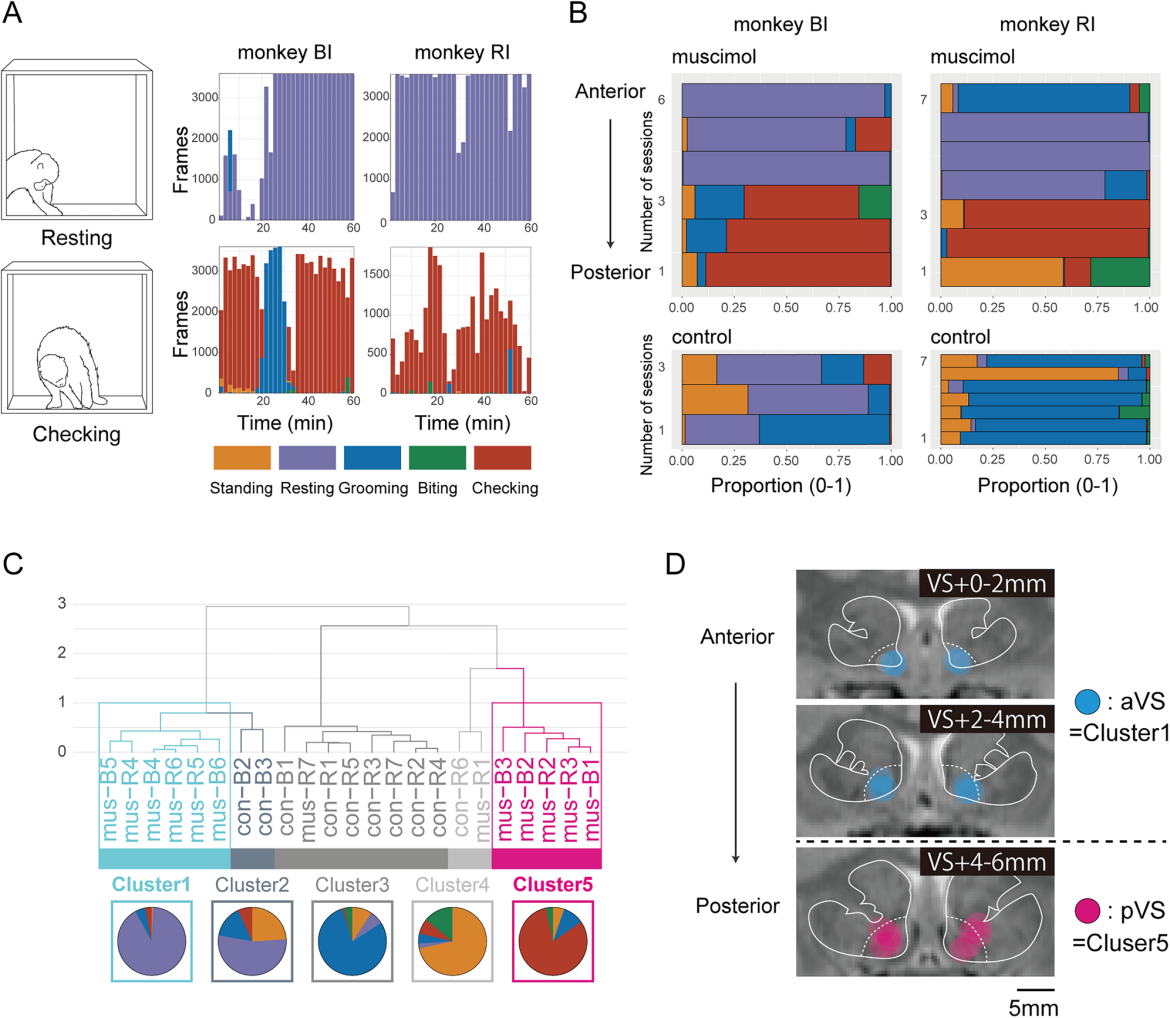
VS inactivation induced location-specific behaviors in the cage. ***A***, Representative behaviors were observed in the cage during the muscimol injection sessions. Top, “resting” behavior, characterized by the monkeys sitting with their head down and remaining motionless without lying down. Bottom, “checking” behavior, in which the monkeys repetitively pinched at the corners of the cage from various angles, frequently changing the pattern and posture of checking. The histograms illustrate examples of the “resting” (mus-B6 and mus-R5 in Extended Data Table 1-1) and “checking” behaviors (mus-B2 and mus-R2) during the sessions of both monkeys. The horizontal axis indicates time, and the vertical axis denotes the number of frames showing each behavior (orange, “standing”; purple, “resting”; blue, “grooming”; green, “biting”; red, “checking”). ***B***, Proportional distributions of the five observed behaviors across sessions. The muscimol injection sessions (top) are arranged from the anterior to pVS injection sites. The horizontal axis shows the proportions of each behavior, whereas the vertical axis indicates the session ID. ***C***, Hierarchical clustering dendrogram (Ward's method with Euclidean distance) of sessions based on behavioral profiles. The vertical axis shows the clustering distance, whereas the horizontal axis displays the session IDs (mus, muscimol injection session; con, control session; B, Monkey BI; R, Monkey RI; numbers indicate the session ID for each treatment; e.g., “mus-B1” indicates the first muscimol injection session for Monkey BI). The pie charts illustrate the mean behavior proportions within each cluster. ***D***, Injection site mapping on MR images from the two monkeys, aligned along the anterior–posterior axis of the VS with the anterior tip at VS +0. Each circle marks an injection site (estimated muscimol diffusion area), with the color indicating the cluster destination (cyan, Cluster 1; magenta, Cluster 5). Dotted lines indicate VS boundaries within the striatum. The muscimol diffusion remained within the VS in 18 of 26 injections. Cluster 1 corresponds to the aVS, and Cluster 5 corresponds to the pVS, based on the distinct localization patterns along the VS.

“Resting” was characterized by the monkeys sitting motionless with their head down, but not lying down (Fig. 2*A*, top). In contrast, “checking” involved repetitive pinching at the corners of the cage, accompanied by a series of varied movements and postural changes such as sitting and standing (Fig. 2*A*, bottom). This behavior differed from simple movement deficits such as motor tics. Notably, “checking” may have been accompanied by negative emotions because the monkeys also displayed threatening behaviors toward other monkeys upon returning to their home cages. Moreover, these inactivation-induced behaviors of “resting” and “checking” were absent when the experimenter was present or visible, such as during transfer to the test cage.

To link the injection sites with spontaneous behaviors, we analyzed video recordings using a deep learning algorithm (YOLO; see Materials and Methods). Following inactivation of aVS, “resting” was predominantly observed (Fig. 2*A*, purple bars). By contrast, “checking” appeared more frequently after inactivation of pVS (Fig. 2*A*, red bars). These site-dependent effects on behavior were consistent between sessions and monkeys and showed a clear distinction from those in control sessions (Fig. 2*B*). Although Monkey BI occasionally displayed postures resembling “resting” in control sessions, these were often accompanied by grooming or other behaviors and clearly differed from sustained “resting” observed during aVS inactivation sessions. A data-driven clustering analysis further validated these site-specific behavioral effects, identifying five behavioral clusters (Fig. 2*C*). Clusters 1 and 5, representing resting- and checking-dominant sessions, respectively, corresponded exclusively to muscimol sessions (Fig. 2*C*, bottom). The other three clusters consisted mainly of control sessions, with two exceptions: the sessions of muscimol injections into the most anterior and posterior sites in Monkey RI (mus-R7 and mus-R1), which were categorized into Clusters 3 and 4, respectively. The injection sites corresponding to Cluster 1 were located in the aVS (0–4 mm from the anterior tip; Fig. 2*D*, cyan), whereas those for Cluster 5 were located in the pVS (4–6 mm from the anterior tip; Fig. 2*D*, magenta). The functional heterogeneity of proportions of “resting” and “checking” behaviors emerged along the anterior–posterior axis of the primate VS with a clear boundary (4 mm from the anterior tip; VS +4 mm), which cannot be explained by chance in either monkey (*χ*^2^ test, *χ*^2^(1) > 161,096; *p* < 1.0 × 10^−16^). These results indicate a significant site-dependent inactivation effect on spontaneous behaviors and thus refer to these two regions as the aVS and pVS.

### Effects of VS inactivation on goal-directed behaviors

To examine the effects of local VS inactivation on goal-directed behaviors, we tested the monkeys using the reward-size task (Fig. 1*C*). In this task, the monkeys released a bar within 1 s after the color change of the fixation point to obtain a liquid reward (1, 2, 4, or 8 drops), which was cued at the beginning of each trial. If they released the bar incorrectly—either too early (early errors) or too late (late errors)—the same stimulus–reward pair was repeated in the subsequent trial, without the option to skip any undesired reward conditions.

Similar to the effects observed in the free-moving context, VS inactivation in the goal-directed context induced site-specific atypical behaviors (“resting” or “checking”) and interfered with task performance. In control sessions, monkeys typically released the bar with minimal hand movements. However, after pVS inactivation, superfluous hand movements unrelated to the task sequence were made, leading to increased overall errors. In contrast, aVS inactivation caused the monkeys to close their eyes and cease performing the task for several to tens of minutes.

**Figure 3.**
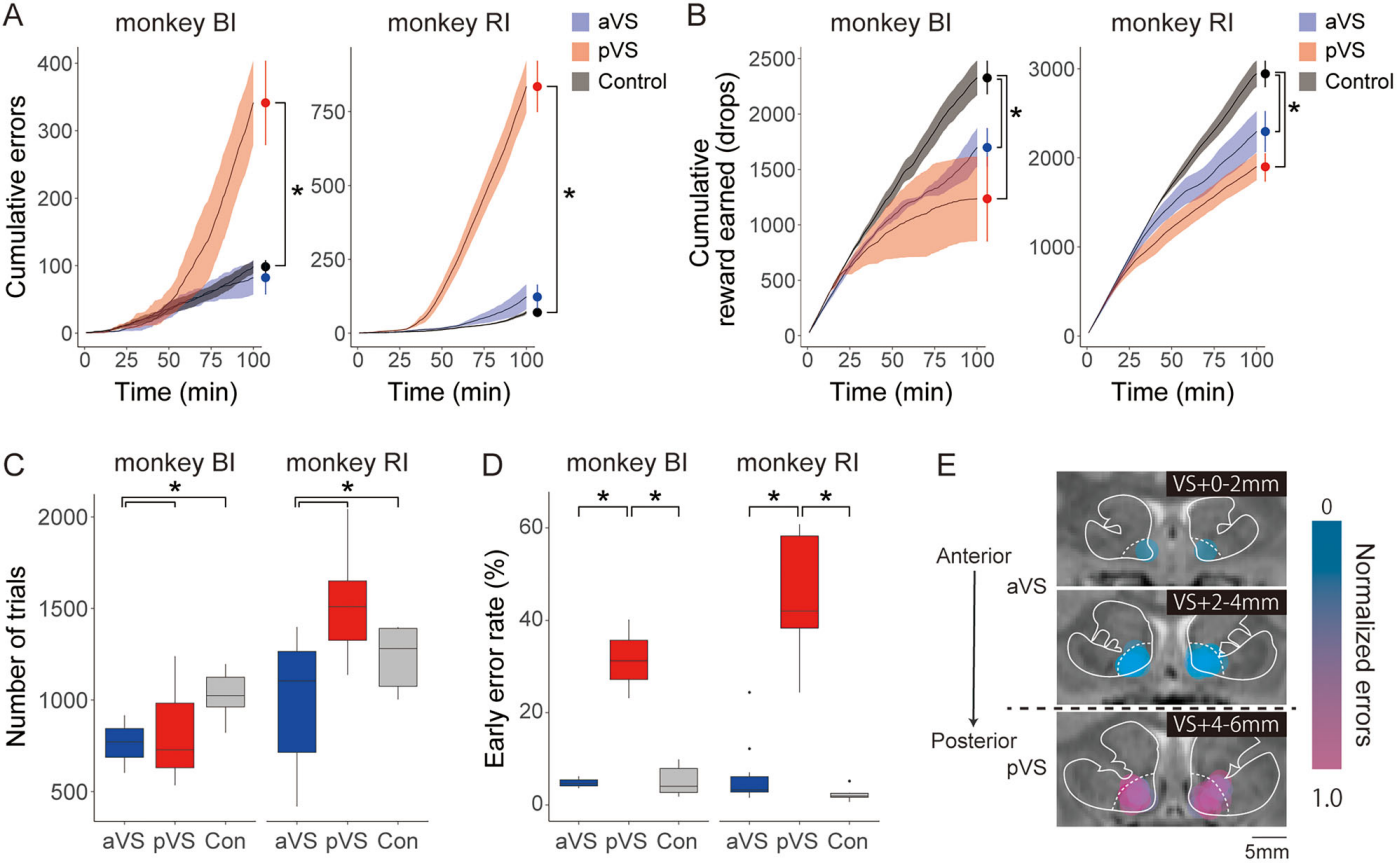
Effects of local VS inactivation in the reward-size task. ***A***, Cumulative error counts over time for each treatment in the reward-size task. Data for Monkey BI (left) and Monkey RI (right) are shown as the mean ± standard error of the mean, with different treatments indicated by color: the aVS in blue, the pVS in red, and the control in gray. Asterisks denote significant differences between treatments (*p < 0.05, one-way analysis of variance with post hoc Tukey's honestly significant difference test). ***B***, Cumulative reward earning over time across treatments (mean ± standard error of the mean). ***C***, The total number of trials initiated under each treatment condition. ***D***, Early error rates for each treatment condition. Center lines indicate means, box limits indicate the first and third quartiles, and whiskers extend from minimum to maximum values. ***E***, Injection sites (estimated muscimol diffusion area) are plotted on magnetic resonance images, with each site indicated by a colored circle. Dotted lines indicate VS boundaries within the striatum. In 33 of 52 injections, the muscimol diffusion remained within the VS. The color gradient from blue to red indicates the normalized error rate per session, with values from 0 (blue) to 1 (red).

We analyzed the temporal dynamics of these atypical behaviors and their relationship with reward accumulation over time. At the beginning of the aVS inactivation sessions, errors progressively increased; this pattern was similar to that of the control sessions (Fig. 3*A*, blue and black, respectively) and to the typical patterns observed in normal monkeys (Minamimoto et al., 2009). Approximately 30 min later, however, the monkeys started to intermittently rest and stop performing the task; this slowed their rate of reward accumulation (Fig. 3*B*, blue) and led to significantly fewer total rewards earned compared with those in the control sessions (one-way ANOVA, *F*_(2,39)_ = 14.6; *p* = 1.97 × 10^−5^; post hoc, aVS vs control, *p* = 0.044; pVS vs control, *p* = 2.8 × 10^−4^). Nonetheless, the monkeys occasionally resumed the task and performed it correctly, indicating that they did not abandon the task entirely.

In contrast to aVS injection, pVS injection caused a rapid increase in error trials at ∼30 min after the beginning of the session (Fig. 3*A*, red), resulting in a significantly higher total number of errors compared with those in the control sessions (one-way ANOVA, *F*_(2,39)_ = 122.6; *p* = 2.0 × 10^−6^; post hoc, *p* = 1.22 × 10^−13^). Despite inefficient task performance, the monkeys continued to engage in the task, albeit with slower accumulation of rewards in the later part of the session. Consequently, their total rewards remained significantly lower than those of controls (Fig. 3*B*, red; post hoc, pVS vs control, *p* = 2.8 × 10^−4^). Together, these results suggest that reward drive was preserved even with VS inactivation, because the monkeys did not completely abandon the task.

These site-dependent profiles of VS inactivation were further validated; inactivation of aVS, but not pVS, significantly reduced the total number of trials initiated (Fig. 3*C*; one-way ANOVA, *F*_(2,39)_ = 7.804; *p* = 0.0015; post hoc, aVS vs control, *p* = 0.041; pVS vs control, *p* = 0.32). Conversely, pVS inactivation increased the number of premature responses, as indicated by a significantly increased ratio of early errors (Fig. 3*D*; one-way ANOVA, *F*_(2,39)_ = 92.12; *p* = 2.67 × 10^−15^; post hoc, *p* = 1.33 × 10^−13^). The observed anterior–posterior differences in total errors (Fig. 3*E*) further emphasized the dichotomy of the inactivation effects.

When the pVS was inactivated unilaterally (left VS +5.25 mm in Monkey BI), the characteristic “checking” behavior observed during bilateral inactivation was absent in the free-moving context. Similarly, in the goal-directed task, unilateral inactivation did not result in the significant increase in errors that was typically observed in the latter part of the session. Although a higher early error rate (51.1%) was noted in unilateral pVS inactivation compared with control conditions, this effect did not substantially disrupt overall performance because the cumulative error count remained relatively low (unilateral pVS, 188 errors; bilateral pVS, 721 ± 90.2 errors; mean ± standard error of the mean). These findings suggest that the behavioral changes described earlier, including “checking” and increased error rates, likely require bilateral VS inhibition to fully manifest.

### Effects of VS inactivation and drive shift

**Figure 4.**
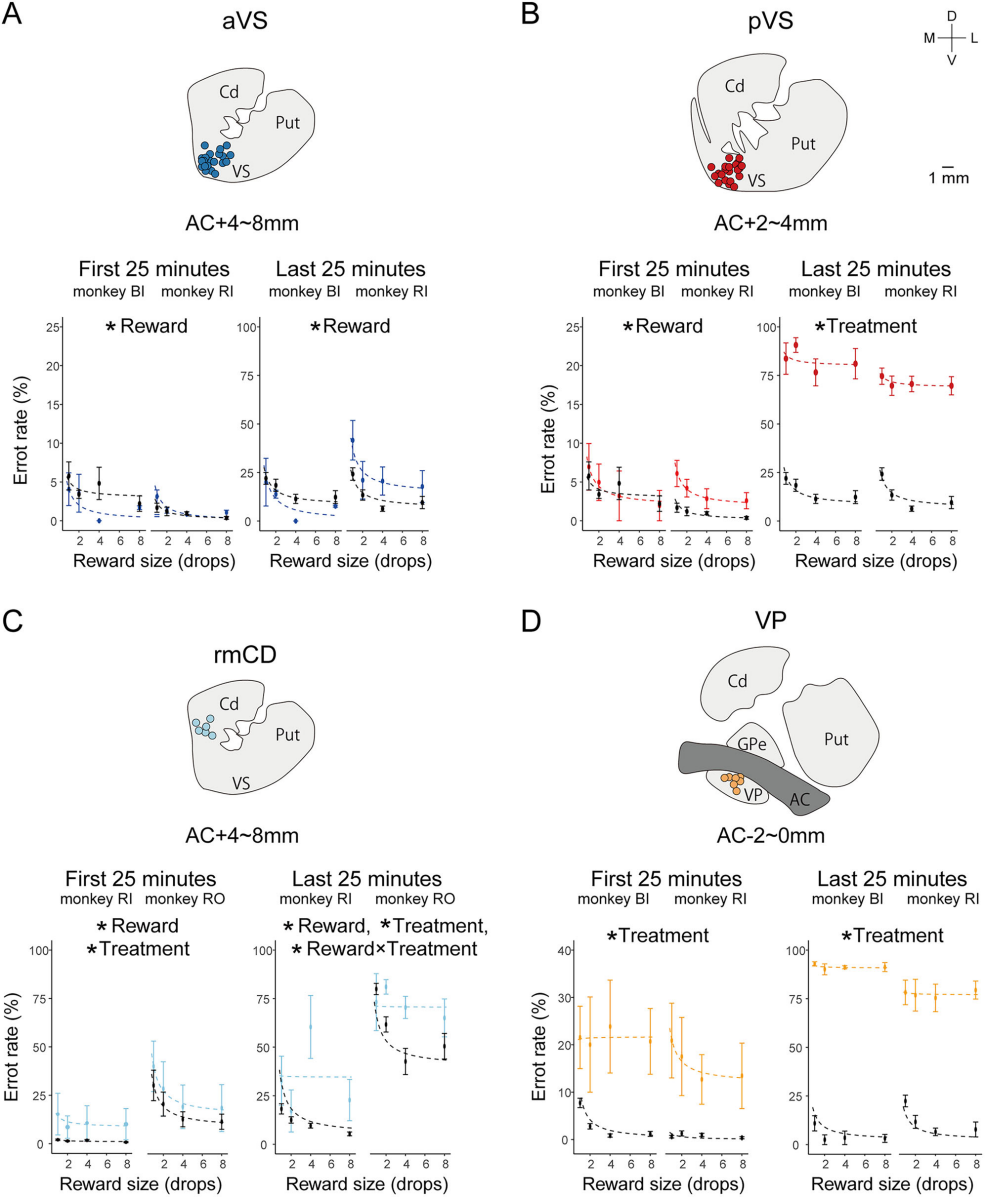
Changes in goal-directed behaviors during the early and late phases of the task. Top row (***A***–***D***), Injection sites for each inactivation condition are displayed on anatomical images. Bottom row (***A*–*D***), Error rates for each reward size (1, 2, 4, and 8 drops) during the first (left) and last (right) 25 min of the task. The results for each inactivation condition are shown below. ***A***, aVS, in blue. ***B***, pVS, in red. ***C***, Rostromedial caudate (rmCD), in cyan. ***D***, Ventral pallidum (VP) is yellow. Control sessions are shown in black. Each dot indicates the mean error rate, with error bars indicating the standard error of the mean. Dotted curves indicate the best-fit inverse function for each condition. Asterisks indicate significant main effects or interaction (*p < 0.05, two-way analysis of variance with post hoc Tukey's honestly significant difference test). AC, anterior commissure; Cd, caudate; GPe, external segment of globus pallidus; Put, putamen; VP, ventral pallidum.

Given the significant effects of VS inactivation on goal-directed behavior, we sought to determine whether these effects stemmed from altered motivation or from changes in specific components of motivation, such as incentives or drive. Notably, we observed that behavioral effects became prominent ∼30 min after the session onset, regardless of the location of VS inactivation. This timing suggests that the emergence of behavioral changes might be related to a shift in the internal drive state because thirst presumably decreased with reward accumulation. To explore this idea, we analyzed error rates during the first and last 25 min of each session, representing the high- and low-drive states, respectively (Fig. 4*A*,*B*).

In the first 25 min of aVS inactivation sessions, error rates remained low and were comparable with those of controls (two-way ANOVA, main effect of treatment, *F*_(1,112)_ = 0.01; *p* = 0.94). There was also a significant main effect of reward size, with an inverse relationship between the reward size and errors (Fig. 4*A*, bottom left; *F*_(3,112)_ = 10.56; *p* = 0.042), consistent with the findings of previous studies using this task (Minamimoto et al., 2009; Nagai et al., 2016; Fujimoto et al., 2019; Hori et al., 2021). In the last 25 min, the overall error rate increased for both control and aVS inactivation sessions—likely reflecting reduced thirst-driven motivation—but no main effect of treatment or interaction was observed, although the effect of the reward size remained significant (Fig. 4*A*, bottom right; treatment, *F*_(1,112)_ = 0.01; *p* = 0.94; reward size, *F*_(3,112)_ = 12.02; *p* = 0.035; treatment × reward size, *F*_(3,112)_ = 0.99; *p* = 0.504). These results suggest that aVS inactivation does not disturb motivational processing for goal-directed behaviors.

Similarly, pVS inactivation did not alter the error pattern in the first 25 min (Fig. 4*B*, bottom left; treatment, *F*_(1,112)_ = 3.098; *p* = 0.33; reward size, *F*_(3,112)_ = 10.09; *p* = 0.045; treatment × reward size, *F*_(3,112)_ = 7.78; *p* = 0.063). However, in the last 25 min, the error rates drastically increased specifically in pVS sessions regardless of the reward size, resulting in a significant main effect of treatment (Fig. 4*B*, bottom right; two-way ANOVA, treatment, *F*_(1,112)_ = 212; *p* = 0.044; reward size, *F*_(3,112)_ = 8.82; *p* = 0.054; treatment × reward size, *F*_(3,112)_ = 2.8; *p* = 0.21). These results suggest that, although motivational processing initially remained intact following pVS inactivation, a progressive disruption of motivational control occurred as reflected by increased errors over time.

The temporal dynamics observed in the present study suggest that the delayed onset of task-relevant behavior likely reflects a shift in each monkey's internal drive. Early in the task, when thirst drive dominated, competing drives such as resting or exploration were suppressed, allowing for goal-directed behavior. As thirst diminished, these competing drives became more prominent, leading to the emergence of task-irrelevant behaviors. However, an alternative explanation involves muscimol pharmacodynamics; it may be that the initial effects of muscimol are too mild to immediately disrupt task performance.

To address the possible mechanisms underlying the above findings more closely, we conducted additional muscimol injections in regions adjacent to the aVS and pVS: the rostromedial caudate (rmCD), located dorsally to the aVS (Fig. 4*C*, top), and the ventral pallidum (VP), located 2–4 mm caudally to the pVS (Fig. 4*D*, top). rmCD inactivation produced significantly higher error rates within the initial 25 min compared with controls (Fig. 4*C*, bottom left; two-way ANOVA, treatment, *F*_(1,199)_ = 8.47; *p* = 0.004; reward size, *F*_(3,199)_ = 3.013; *p* = 0.0312). In the last 25 min, error rates further increased, and a significant interaction between the reward size and treatment emerged (Fig. 4*C*, bottom right; two-way ANOVA, treatment, *F*_(1,199)_ = 31.05; *p* = 3.12 × 10^−8^; reward size, *F*_(3,199)_ = 7.51; *p* = 8.75 × 10^−5^; treatment × reward size, *F*_(3,199)_ = 5.0; *p* = 0.0023). Similarly, VP inactivation produced significant effects in both the initial and final 25 min, in which the main effect of the reward size disappeared and a significant main effect of treatment emerged (two-way ANOVA, first 25 min, treatment, *F*_(1,71)_ = 46.28; *p* = 2.69 × 10^−9^; reward size, *F*_(3,71)_ = 0.35; *p* = 0.79; last 25 min, treatment, *F*_(1,71)_ = 1,212.51; *p* = 2.28 × 10^−46^; reward size, *F*_(3,71)_ = 2.55; *p* = 0.063). These results indicate that muscimol injections can induce behavioral changes immediately after the session onset, suggesting that the delayed effects observed with aVS and pVS inactivation are unlikely to be caused by muscimol kinetics or spatial diffusion.

Together, these results support the idea that the delayed emergence of task-irrelevant behaviors following aVS and pVS inactivation likely arose because of a diminished thirst drive, thus allowing other desires (such as rest and exploration) to become more influential. Furthermore, the marked impact of rmCD and VP inactivation on motivational value—specifically, the altered relationship between reward size and error rate—emphasizes the importance of these regions in incentive processing. This finding contrasts with the effects observed with VS inactivation and underscores the unique roles of the VS in both processing multiple drives and regulating behaviors according to the balance among these drives.

Finally, we examined the effects of VS inactivation on other behavioral indices. There was no significant treatment effect on reaction time for either the first or last 25 min (Fig. 4*B*; two-way ANOVA, first 25 min, treatment, *F*_(2,168)_ = 10.68; *p* = 0.086; reward size, *F*_(3,168)_ = 14.96; *p* = 0.026; last 25 min, treatment, *F*_(2,168)_ = 0.21; *p* = 0.83; reward size, *F*_(3,168)_ = 7.67; *p* = 0.064). These results further suggest that the VS does not play a direct role in the initiation of goal-directed action.

### Distinct cortical and subcortical inputs to the aVS and pVS revealed by retrograde tracing

**Figure 5.**
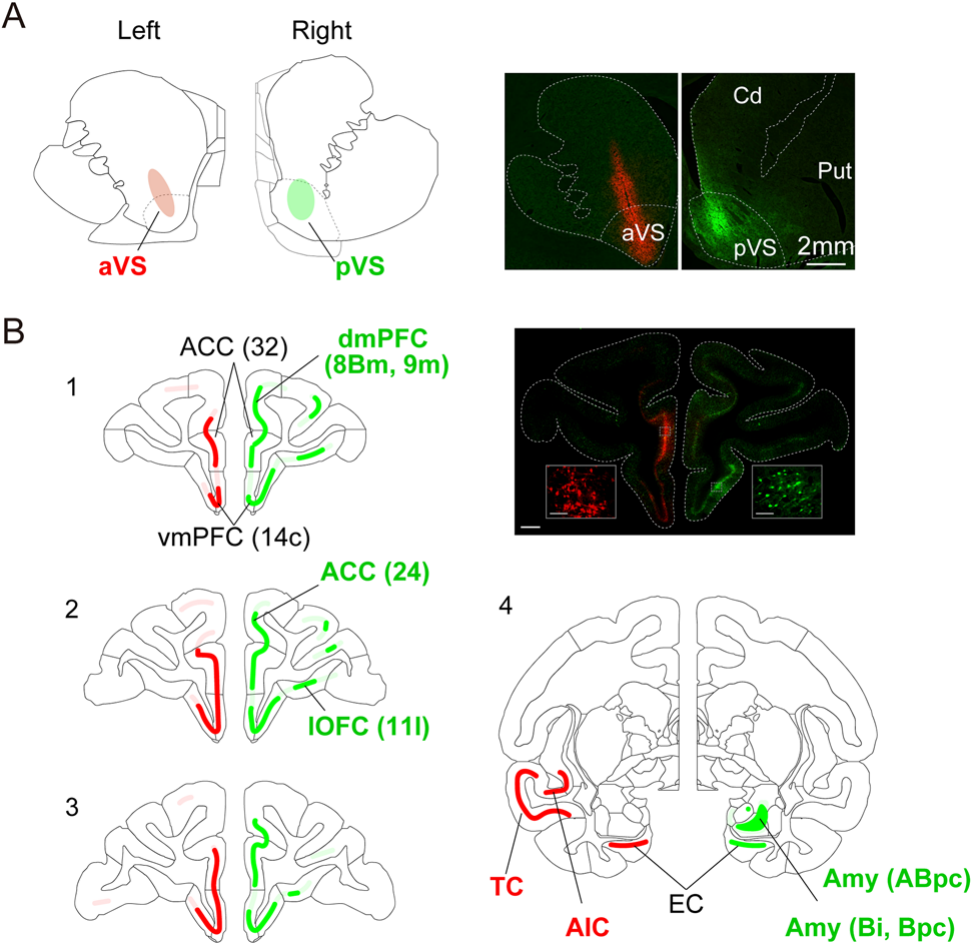
Anatomical projection patterns to the aVS and pVS. ***A***, Schematic illustration of the injection sites of retrograde viral vectors in the aVS and pVS (left) alongside a fluorescently stained image (right). In a single monkey (Monkey #250), AAV2retro-hSyn-mScarlet was injected into the aVS of the left hemisphere, and AAV2retro-hSyn-AcGFP was injected into the pVS of the right hemisphere. ***B***, Coronal sections showing mScarlet and GFP expression along a rostral to caudal gradient, with sections labeled 1–4. These panels represent sections at different anterior–posterior coordinates (1, AC +18.45 mm; 2, AC +16.2 mm; 3, AC +14.4 mm; and 4, AC +0.45 mm). Regions expressing mScarlet and GFP are shaded in red and green, respectively, with the color intensity qualitatively indicating expression strength. The text colors correspond to mScarlet (red) and GFP (green) expression; the black text denotes regions expressing both markers. The fluorescently stained image in the top right corresponds to Schematic 1 (scale bar, 2 mm), and the inset shows an example of retrogradely labeled neurons expressing the fluorescent proteins (scale bar, 100 µm). ACC, anterior cingulate cortex; AIC, anterior insular cortex; Amy, amygdala; ABpc, accessory basal nucleus of the amygdala; parvicellular division; Bi, basal nucleus of the amygdala, intermediate subdivision; Bpc, basal nucleus of the amygdala, parvicellular subdivision; dmPFC, dorsomedial prefrontal cortex; EC, entorhinal cortex; lOFC, lateral orbitofrontal cortex; TC, temporal cortex; vmPFC, ventromedial prefrontal cortex.

The finding that aVS and pVS inactivation induced different atypical behaviors with a clear functional boundary between these regions suggests that each region is a part of distinct neural circuits that differentially controls behavior. To investigate this concept, we performed a retrograde tracer study to map the cortical and subcortical connections that are specific to the aVS and pVS regions. AAV2retro-hSyn-mScarlet and AAV2retro-hSyn-AcGFP vectors were injected into the left aVS (VS +3 mm) and right pVS (VS +5 mm), respectively, in an additional monkey (Monkey #250). The injection sites were confirmed by observing localized fluorescent signals within the intended regions, without any overlap along the anterior–posterior axis; however, we noted minor leakage into the caudate nucleus, which is dorsal to the aVS (Fig. 5*A*).

The retrogradely labeled neurons of the aVS (red) and pVS (green) were mapped onto a macaque atlas (Fig. 5*B*). Both regions commonly received projections from the medial prefrontal cortex, including its ventral part (Areas 10mc, 14c, 14r, and 32), and the entorhinal cortex (Fig. 5*B*1–3). In contrast, in the aVS only, labeled neurons were also identified in the anterior insular cortex and temporal cortex (Fig. 5*B*4, left). In addition, retrogradely labeled neurons of the pVS were selectively observed in the lateral orbitofrontal cortex (Area 11l), dorsomedial prefrontal cortex (Areas 8Bm and 9 m), and dorsal anterior cingulate cortex (Area 24c; Fig. 5*B*1–3, right), as well as in the basal nucleus and accessory basal nucleus of the amygdala (Fig. 5*B*4, right).

In terms of anterograde projections, axon terminals from both the aVS and pVS were labeled in several brain regions, including the ventral pallidum, ventral tegmental area, and internal segment of the globus pallidus. Visual inspection of fluorescence labeling revealed no clear differences in projection patterns between the aVS and pVS in these brain regions, suggesting that some output pathways may be shared between the two VS regions.

These anatomical results support the hypothesis that the aVS and pVS form distinct neural circuits, especially in terms of their origins of projection. The “resting” behavior induced by aVS inactivation may be controlled by regions uniquely connected to the aVS, such as the anterior insula, whereas the “checking” behavior induced by pVS inactivation may be mediated by regions specifically connected to the pVS, including the lateral orbitofrontal cortex and amygdala. This distinct connectivity between the aVS and pVS aligns with the functional heterogeneity observed between the two regions, thus highlighting their specialized roles in motivational and behavioral regulation.

## Discussion

In the present study, we examined the effects of inactivating mirror-symmetrical VS regions on free-moving and goal-directed behaviors in macaque monkeys. We revealed that the primate VS is functionally heterogeneous, comprising distinct aVS and pVS regions. Inactivating these regions resulted in two specific behaviors: “resting” and “checking.” “Resting” was characterized by a low-activation state, whereas “checking” resembled stereotyped, compulsive-like behaviors rather than a motor disorder. These behaviors were not observed during unilateral inactivation, suggesting that bilaterality is crucial for the manifestation of these region-specific effects. Notably, despite these behavioral changes, VS inactivation did not affect incentive processes or reward drives in goal-directed tasks. Furthermore, retrograde tracing experiments demonstrated that the aVS and pVS have distinct neural connections, indicating that these regions form separate corticostriatal circuits. Our findings suggest that the aVS and pVS are critical for regulating intrinsic drives, thus orienting the organism toward appropriate/impending behaviors. The inactivation of each region appears to elicit specific behavioral repertoires, reflecting their functional specialization.

The primate VS is generally considered to be associated with motivational control, particularly in terms of reward-driven behaviors; numerous electrophysiological and neuroimaging studies emphasize the neural correlates of the VS in reward expectation and rewarding events (Schultz et al., 1992; Bowman et al., 1996; Hollerman et al., 1998; Shidara et al., 1998; Tremblay et al., 1998; Knutson et al., 2000, 2001, 2005; Cromwell and Schultz, 2003; Botvinick et al., 2009; Croxson et al., 2009; Nakamura et al., 2012). However, primate lesion studies suggest that the VS may not be a center for reward-driven behavior (Stern and Passingham, 1996; Costa et al., 2016). This ongoing debate led us to further explore its functions, specifically investigating potential functional differentiation along the anterior–posterior axis, similar to findings in rodents (Reynolds and Berridge, 2001, 2002, 2008).

In our study, we used CT and MR imaging to precisely inject muscimol into bilateral and symmetrical regions of the VS while minimizing the effects on nearby reward-related brain regions. We examined the causal role of the VS under two different motivational conditions. Using a task that allowed us to separate two motivational factors—incentive value and reward drive—we demonstrated that VS inactivation led to the emergence of nonreward-dependent atypical behaviors such as “resting” and “checking” without impairing incentive value or reward drive in the goal-directed task. This suggests that the VS may not regulate goal-directed behavior solely based on reward value or internal drive but may instead play a critical role in controlling and suppressing various motivation types. VS inactivation appeared to release these superfluous behaviors, which interfered with goal-directed activity.

In free-moving contexts, monkeys showed atypical behaviors (“resting” or “checking”) throughout the entire session. However, in goal-directed tasks, these behaviors only emerged after 30 min, raising questions about the underlying mechanisms. Our comparative analysis of previous data from the inactivation of VS-adjacent regions, the rmCD and VP (Nagai et al., 2016; Fujimoto et al., 2019), clearly indicated that the effects appeared immediately after task initiation. This suggests that the delayed appearance of atypical behaviors following VS inactivation is unlikely to be caused by a slow pharmacological onset. Instead, it is more likely that an initially high reward drive suppresses other competing drives such as rest, which become prominent after the reward drive diminishes. This temporal pattern contrasts with the directly impaired incentive-based behaviors from task initiation with rmCD and VP inactivation. These findings suggest that while the rmCD and VP are directly involved in incentive motivation, the VS may play a broader regulatory role by suppressing competing, nonreward-driven motivations.

One limitation of our study is the potential spread of muscimol beyond the intended regions of the VS. Although the potential effects on nearby regions cannot be completely ignored when estimating the spread of muscimol (2–3 mm in diameter for a 2 µl; Tremblay et al., 2009; Murata et al., 2015), they remained within the VS in most of the injections (Figs. 1*A*, 2*D*, 3*E*). Furthermore, the distinct and consistent behaviors that we observed, which were clearly associated with locations along the VS anterior–posterior axis, suggest that muscimol spread was likely confined to the target regions. The absence of any intermediate or mixed behaviors at the aVS/pVS border further supports this conclusion.

The series of studies on rodent nucleus accumbens conducted by Berridge and colleagues offer important insights into the functions of the VS in primates (Reynolds and Berridge, 2001, 2002; Richard et al., 2013; Castro and Berridge, 2014; Baumgartner et al., 2020). These earlier studies demonstrated that muscimol inactivation of the rostral and caudal regions of the nucleus accumbens medial shell produce opposing motivational behaviors—appetitive eating and defensive treading for the rostral and caudal shell, respectively. Although these positive and negative motivational behaviors are different from the “resting” and “checking” behaviors observed in our study, they may reflect species-specific differences in the expression of motivational states.

The neuropsychological mechanisms driving the atypical behaviors induced by aVS and pVS inactivation remain key to interpreting our findings. The “resting” behavior observed after aVS inactivation resembles sleep in that monkeys became motionless. However, typical sleep behaviors, such as lying down, were not noted, and monkeys only rested when the experimenter was absent; this suggests a more voluntary, controlled resting state rather than homeostatic sleep. This behavior is reminiscent of hypoactivity with preserved executive function, as observed with unilateral pharmacological activation of the Monkey VS (Worbe et al., 2009).

In contrast, the “checking” behavior induced by pVS inactivation involved repetitive actions that were distinct from motor disturbances such as tics. This behavior was akin to compulsive grooming in rodents, which is elicited by the activation of excitatory inputs to the VS from the orbitofrontal cortex and midbrain dopamine neurons (Ahmari et al., 2013; Xue et al., 2022). Comparable behaviors have been reported in primates following the manipulation of specific brain regions (Grabli et al., 2004; Worbe et al., 2009; Rotge et al., 2012; Saga et al., 2022). The repetitive nature of “checking” may also serve as a new model of OCD in humans. Although compulsive behaviors in clinical settings vary, ranging from washing and cleaning to checking, the “checking” behaviors observed in the present study represent a form of stereotyped behavior that has not previously been reported and may offer new insights into the neural mechanisms of OCD.

The concept of a “security motivation system,” proposed by Szechtman and Woody (2004), may explain the neuropsychological basis of compulsive-like behaviors observed in this study. According to this model, behaviors such as checking are driven by an inability to achieve a “feeling of knowing” that the environment is secure, leading to compulsive behavior. The pVS may be a central component of such a security motivation system, and its impairment might explain the emergence of compulsive-like behaviors.

Our anatomical tracing study suggests that while the VS projection patterns were largely consistent with previous findings (Haber, Lynd, et al., 1990; Haber, Wolfe, et al., 1990; Gimenez-Amaya et al., 1995; Haber et al., 1995, 2000, 2006; Chikama et al., 1997; Fudge and Haber, 2002; Fudge et al., 2002, 2004; Fudge and Tucker, 2009), aVS and pVS form distinct corticosubcortical circuits. This adds finer granularity to previously reported anterior–posterior distinctions in corticolimbic striatal projections, particularly those contrasting the VS with more posterior limbic regions such as the caudate tail (McHale et al., 2022). These distinctions provide potential insights into conditions such as apathy and OCD, as observed in the phenomenological similarities between the behaviors induced by VS inactivation and these symptoms. The low-activity “resting” state induced by aVS inactivation may be related to symptoms of apathy, particularly the “autoactivation deficit” described in human patients (Levy and Dubois, 2006). Interestingly, the insular cortex, which sends selective projection outputs to the aVS, reportedly shows atrophy in patients with apathy (Moon et al., 2014). Moreover, the “checking” behavior observed with pVS inactivation mirrors the compulsive behaviors commonly seen in OCD. Human imaging studies of OCD patients have revealed activation in the lateral orbitofrontal cortex and amygdala (Rotge et al., 2010; Simon et al., 2010), both of which send selective outputs to the pVS. Notably, clinical studies have reported that the targets of DBS for treatment-resistant OCD patients shifted to posterior regions of VS (Greenberg et al., 2010). These behavioral circuit parallels suggest that further elucidation of the neural mechanisms centered on the aVS and pVS may enhance our understanding of the underlying mechanisms of clinical conditions, such as apathy and OCD, and might lead to the identification of effective treatment targets.

In conclusion, the present study demonstrates that the aVS and pVS play distinct roles in regulating nonreward-dependent behaviors, such as “resting” or “checking,” which emerge following region-specific inactivation. Our findings suggest that the VS not only governs reward-driven actions but also suppresses competing motivations, thereby underscoring its broader role in behavioral regulation. The similarities between these behaviors and the symptoms observed in apathy and OCD suggest the existence of similar underlying neural mechanisms, thus offering new insights into potential therapeutic targets for psychiatric conditions.
